# A visual dataset for recognition of rice varieties

**DOI:** 10.1016/j.dib.2024.110442

**Published:** 2024-04-18

**Authors:** Md. Masudul Islam, Galib Muhammad Shahriar Himel, Mohammad Shorif Uddin, Md. Golam Moazzam

**Affiliations:** a Department of Computer Science and Engineering, Jahangirnagar University, Dhaka, Bangladesh; b Department of Physics, Jahangirnagar University, Dhaka, Bangladesh

**Keywords:** Image classification, Rice varieties, Computer vision, Deep learning

## Abstract

This article presents a comprehensive dataset sourced from various markets across Bangladesh, highlighting 20 distinct rice varieties predominantly consumed locally. The dataset encompasses a diverse range of rice strains, including Subol Lota, Bashmoti (Deshi), Ganjiya, Shampakatari, Sugandhi Katarivog, BR-28, BR-29, Paijam, Bashful, Lal Aush, BR-Jirashail, Gutisharna, Birui, Najirshail, Pahari Birui, Polao (Katari), Polao (Chinigura), Amon, Shorna-5, and Lal Binni. Using a smartphone camera, low-resolution images capturing the essence of each rice variety were meticulously obtained, resulting in a total of 4,730 images with a non-uniform distribution. The dataset also includes augmented data, totaling 23,650 images. This precisely curated dataset holds significant promise and utility, showcasing diverse attributes, including the unique representation of 20 rice varieties, each characterized by distinct colors, sizes, and potential applications within the agricultural sector.

Specifications Table

**Table:** 

Subject	Computer science
Specific subject area	Artificial Intelligence, Computer Vision and Pattern Recognition
Type of data	Image
Data collection	This dataset showcases a compilation of low-resolution images featuring 20 well-known rice varieties sourced from diverse locations across Bangladesh. The rice samples were meticulously collected from both rural areas and marketplaces, providing a comprehensive and varied representation. Serving as a visual compendium, the dataset offers a thorough insight into the distinct characteristics of these rice varieties, facilitating precise classification. It encompasses 20 distinct classes, including *Subol Lota, Bashmoti (Deshi), Ganjiya, Shampakatari, Sugandhi Katarivog, BR-28, BR-29, Paijam, Bashful, Lal Aush, BR-Jirashail, Gutisharna, Birui, Najirshail, Red Cargo, Polao (Katari), Polao (Chinigura), Amon, Shorna-5, and Lal Binni*, totaling 4730 original JPG format images and 23,650 augmented images. These images were captured using an iPhone 11 camera with a 5x zoom feature. Each image capturing these rice varieties was diligently taken between October 18 and November 29, 2023.
Data source location	**Location:** Dinajpur and Dhaka **Country:** Bangladesh **Latitude and longitude:** Place Between 23° 47′ 44.178′' N 90° 21′ 13.151′' E 25° 37′ 40.487′' N 88° 37′ 59.444′' E
Data accessibility	**Repository name:** Mendeley Data **Data identification number:**10.17632/3mn9843tz2.3 **Direct URL to data:**https://data.mendeley.com/datasets/3mn9843tz2

## 1 Value of the Data

• Valuable for farmers, breeders, and food scientists, aiding in tasks such as selecting seeds, maintaining varietal integrity, and optimizing storage and processing conditions for different rice types.
• Beneficial for agronomists and agricultural scientists aiming to enhance crop cultivation practices, as the dataset provides a visual reference for various rice varieties and their unique features.
• Valuable for data scientists and machine learning practitioners interested in image classification, as the dataset serves as a robust training and testing resource for developing models to automatically classify different rice varieties.
• The AI model developed using this comprehensive rice dataset will serve as a powerful tool to enable ordinary consumers to promptly identify various rice varieties using their smartphones. This will help combat the blending of different rice types by unscrupulous traders, ensuring transparency in the marketplace and during sales transactions.
• Can be used as a standard reference for researchers developing new image classification algorithms for objects resembling grains. Offering a standardized dataset enables fair comparisons between different methodologies. Given the complexity of distinguishing among 20 similar rice types, this dataset aids in the development of more resilient algorithms for intricate image classification tasks.

## 2 Background

Rice, a staple food for over half of the world's population, is a cereal grain that has played a pivotal role in global agriculture and cuisine for thousands of years [1]. Belonging to the grass species Oryza sativa or Oryza Glaberrima, rice is cultivated in a diverse array of environments, ranging from flooded paddies to upland areas. There are numerous varieties (approx. 40,000) of rice, each exhibiting unique characteristics in terms of size, shape, color, and aroma [2]. Popular varieties include Basmati, Jasmine, Arborio, and the various types of short-grain and long-grain rice. This versatile grain serves as a primary food source in many cultures, offering a high-energy staple with essential nutrients such as carbohydrates and proteins. Beyond its nutritional significance, rice cultivation plays a crucial role in the economies of numerous countries, supporting livelihoods and shaping agricultural landscapes. The adaptability of rice to different climates has made it a vital crop for food security globally. Its role in sustaining populations and fostering cultural connections underscores the paramount importance of rice in both global agriculture and human societies.

Recently various types of rice bran have been genetically engineered to populate the market with lots of rice varieties making it even harder than before to distinguish among different varieties it is very necessary to identify them accurately because even though there are some similarities among some species, they have varying nutrition and production factors. Traditionally, identification relied on expertise from domain specialists, a process prone to time constraints and occasionally hindered by the scarcity of experts. However, with the advent of artificial intelligence, this task can now be executed with remarkable precision using intelligent systems. A crucial prerequisite for training these intelligent systems is access to credible data. As deep learning is distinguished by its capacity to comprehend diverse patterns and characteristics within datasets through training and subsequent classification. When machine learning algorithms are applied to extensive datasets, they demand more training time yet yield superior outcomes. However, assessing algorithm performance on smaller datasets poses challenges due to limited data availability. Therefore, datasets of moderate size with significant variation can effectively demonstrate the capabilities of algorithms, particularly in challenging tasks.

Identification of rice varieties primarily relies on their distinctive attributes such as shape, color, and texture. However, existing image datasets in this domain suffer from limitations [3]. Some datasets lack an adequate number of rice variants, while others may have abundant data but lack variation in terms of rice characteristics like shape, color, and texture. Consequently, the absence of comprehensive datasets encompassing diverse rice varieties and their characteristic traits poses a significant challenge. Therefore, the development of a robust rice dataset that captures the variations in rice varieties and their traits is imperative to address this gap effectively.

The compilation of this rice varieties image dataset originated from a motivation to bridge gaps in agricultural research and technology. Recognizing the pivotal role of rice as a staple food, the dataset was meticulously curated to address the need for a comprehensive visual resource encompassing diverse grain varieties. Motivated by a commitment to foster advancements in crop science, the dataset aims to empower researchers, agronomists, and technologists with a rich repository of rice images for visual diversity studies, agricultural innovation, and machine learning applications. The context lies in fostering a deeper understanding of rice varieties to enhance food security, agricultural practices, and technological solutions in the evolving landscape of agriculture.

## 3 Data Description

This data article presents a novel and extensive rice grain image dataset encompassing 20 distinct varieties: Subol Lota, Bashmoti (Deshi), Ganjiya, Shampakatari, Sugandhi Katarivog, BR-28, BR-29, Paijam, Bashful, Lal Aush, BR-Jirashail, Gutisharna, Birui, Najirshail, Red Cargo, Polao (Katari), Polao (Chinigura), Amon, Shorna-5, and Lal Binni. We captured the images using a smartphone camera under the guidance of an expert in the agricultural field. The dataset comprises 4730 original images, meticulously captured to represent the unique visual characteristics of each variety. Original images are of high resolution (853 × 853 pixels), while augmented images are optimized for computational efficiency (512 × 512 pixels). Detailed information about the dataset composition and visual features of the rice grains are provided in Table 1, Table 2 respectively. For convenient accessibility, the rice varieties dataset is publicly available on the Mendeley repository [4] in two distinct zip files: “Original.zip” and “Augmented.zip”. Each zip file contains 20 sub-directories, ensuring efficient organization and retrieval of images for specific varieties. We named our dataset ‘**Aruzz22.5K**’. This comprehensive and readily accessible rice grain image dataset paves the way for significant advancements in rice variety identification, machine learning, and computer vision research.

**Table 1:** Rice varieties dataset description.

Sl.	Name	Folder name	Original images (Resolution 853 × 853)	Augmented images (Resolution 512 × 512)
1	**Subol Lota**	1_Subol_Lota	232	1160
2	**Bashmoti**	2_Bashmoti	230	1150
3	**Ganjiya**	3_Ganjiya	233	1165
4	**Shampakatari**	4_Shampakatari	234	1170
5	**Katarivog**	5_Katarivog	233	1165
6	**BR-28**	6_BR28	230	1150
7	**BR-29**	7_BR29	233	1165
8	**Paijam**	8_Paijam	234	1170
9	**Bashful**	9_Bashful	252	1260
10	**Lal Aush**	10_Lal_Aush	231	1155
11	**Jirashail**	11_Jirashail	231	1155
12	**Gutisharna**	12_Gutisharna	256	1280
13	**Red Cargo**	13_Red_Cargo	240	1200
14	**Najirshail**	14_Najirshail	231	1155
15	**Polao (Katari)**	15_Katari_Polao	242	1210
16	**Lal Biroi**	16_Lal_Biroi	231	1155
17	**Polao (Chinigura)**	17_Chinigura_Polao	237	1185
18	**Amon**	18_Amon	250	1250
19	**Shorna-5**	19_Shorna5	238	1190
20	**Lal Binni**	20_Lal_Binni	232	1160
	**TOTAL**		**4730**	**23,650**

**Table 2:** Rice varieties visual features.

Sl.	Image	Name	Features
1		Subol Lota	The Subol Lota rice grain exhibits an elongated and slender shape, boasting a length-to-width ratio of about 3:1. Its smooth, glossy surface is white with a subtle creamy tint, showcasing a translucent quality. Slightly curved, it features a convex ventral surface and a concave dorsal surface, along with a small, dark spot at the hilum, its point of attachment to the rice plant.
2		Bashmoti	The Bashmoti rice grain boasts an elongated and slender form, showcasing a smooth, glossy surface. Its translucent appearance is complemented by a rounded end and a pointed tip, featuring a small, dark spot at the hilum, maintaining a length-to-width ratio of around 3:1.
3		Ganjiya	The Dekhichata red rice is short, and plump, and boasts a length-to-width ratio of around 2:1. Characterized by a slightly rough texture and a visible bran layer, the rice displays a deep, rich red color, rendering it opaque. With a rounded end and tip, this grain's bran layer is rich in nutrients, including fiber, vitamins, and minerals.
4		Shampakatari	Elegant and slender, Shampakatari rice boasts a smooth, glossy surface and a creamy white hue. Its elongated form tapers gracefully to a pointed tip, while a distinct crease runs along its dorsal surface.
5		Katarivog	The Katarivog rice grain is elongated and slender, with a length-to-width ratio of approximately 3:1. The rice grain has a smooth, glossy surface. The rice grain is white in color, with a slight creamy hue. The rice grain has a translucent appearance, with a slight opacity.
6		BR-28	The BR-28 rice grain is compact, exhibiting a length-to-width ratio of around 2:1. It possesses a smooth, shiny surface, appearing white with a subtle creamy tint. Notably, it features a clear longitudinal crease along the dorsal surface.
7		BR-29	Similar to BR-29, the rice grain shares its characteristics, featuring a rounded end and a pointed tip. The grain exhibits a subtle taper from the base to the tip.
8		Paijam	The Paijam rice exhibits a short, stout profile with a mildly textured surface, displaying a noticeable bran layer. Its color is a golden hue, conveying a deep, rich tone, and possesses an opaque quality. The grain concludes with a rounded end and tip, contributing to its distinct visual characteristics.
9		Bashful	The Bashful rice kernel is petite, spherical, and compact. It exhibits a white tone with a subtle creaminess, evident within its core.
10		Lal Aush	The Dekhichata Lal Aush rice is characterized by its short, plump grain. It possesses a slightly textured surface with a visible outer layer, showcasing a red hue that is deep and rich. This outer layer, or bran, is abundant in nutrients, including fiber, vitamins, and minerals.
11		Jirashail	The Jirashail rice variety features an elongated and slender grain with a smooth, glossy surface. It is white with a subtle creamy tint, exhibiting a translucent quality, a rounded end, and a pointed tip.
12		Gutisharna	Gutisharna rice exhibits a compact, plump structure, displaying a light golden-yellow hue with a deep, translucent quality. Its glossy surface, attributed to elevated amylose content, imparts firmness and luster to cooked rice.
13		Red Cargo	The extended red rice kernel is slim and elongated with a noticeable lengthwise crease. Its deep, rich red hue is distinctive, featuring a subtle taper from the base to the tip, more pronounced than in counterparts like Cambodian red rice.
14		Najirshail	Najirshail rice is slim and elongated, possessing a length-to-width ratio of about 3:1. It exhibits a sleek, shiny surface and a creamy white hue. The grain features a rounded end and a pointed tip, with a small, dark spot at the hilum.
15		Polao (Katari)	The Katari Polao rice exhibits a compact, plump morphology with a gentle taper from base to tip, more pronounced than in comparable short-grain rice types like Korean rice. It boasts a rich, creamy white hue, intenser than varieties like Italian rice.
16		Lal Biroi	The rice grain exhibits a subtle elongation, with a length-to-width ratio of around 2:1, slightly surpassing similar characteristics in red rice varieties like Bhutanese and Cambodian red rice. It boasts a vibrant, deep red hue, more vivid than Thai red rice.
17		Polao (Chinigura)	The rice grain displays a gently narrowing form, where the base is marginally broader than the apex. This taper is more evident compared to other slim rice varieties like Basmati. Its hue is a rich, creamy white, surpassing the intensity found in alternative white rice types such as IRRI 6.
18		Amon	The Aman rice is renowned for its compact, stout form and elevated amylose levels. It features a subtly lengthened structure, with a ratio of about 2:1, slightly surpassing short-grain varieties like Japanese rice. The grain boasts a rounded end and tip.
19		Shorna-5	Similar to Gutisharna types but with a slightly yellowish hue, a see-through quality, and a gently tapered shape, featuring a base slightly broader than the tip.
20		Lal Binni	Slightly elongated form, with a length-to-width ratio of about 3:1, rendering it more slender than other red rice types. Features a rounded end, smooth surface, and visible bran layer.

**Fig. 1:**
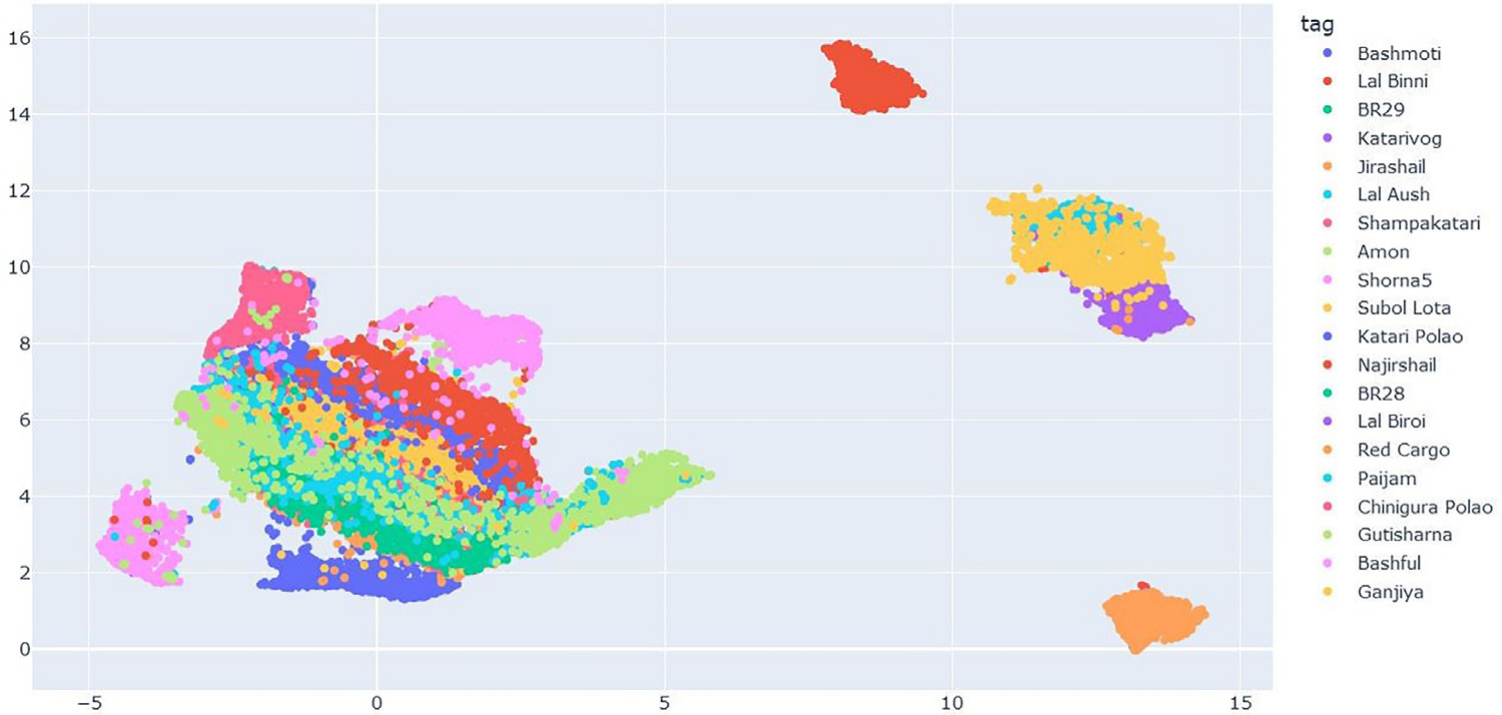
UMAP scatter plot visualization.

Visualizing our image dataset using UMAP allows us to assess whether our image vectors effectively capture features suitable for training a model. A densely clustered UMAP scatter plot indicates a higher level of optimism regarding our model's training effectiveness. Conversely, a scattered plot suggests the need to explore alternative methods for encoding our image data. Fig. 1. Shows the UMAP scatter plot of the dataset.

## 4 Experimental Design, Materials, and Methods

**Fig. 2:**
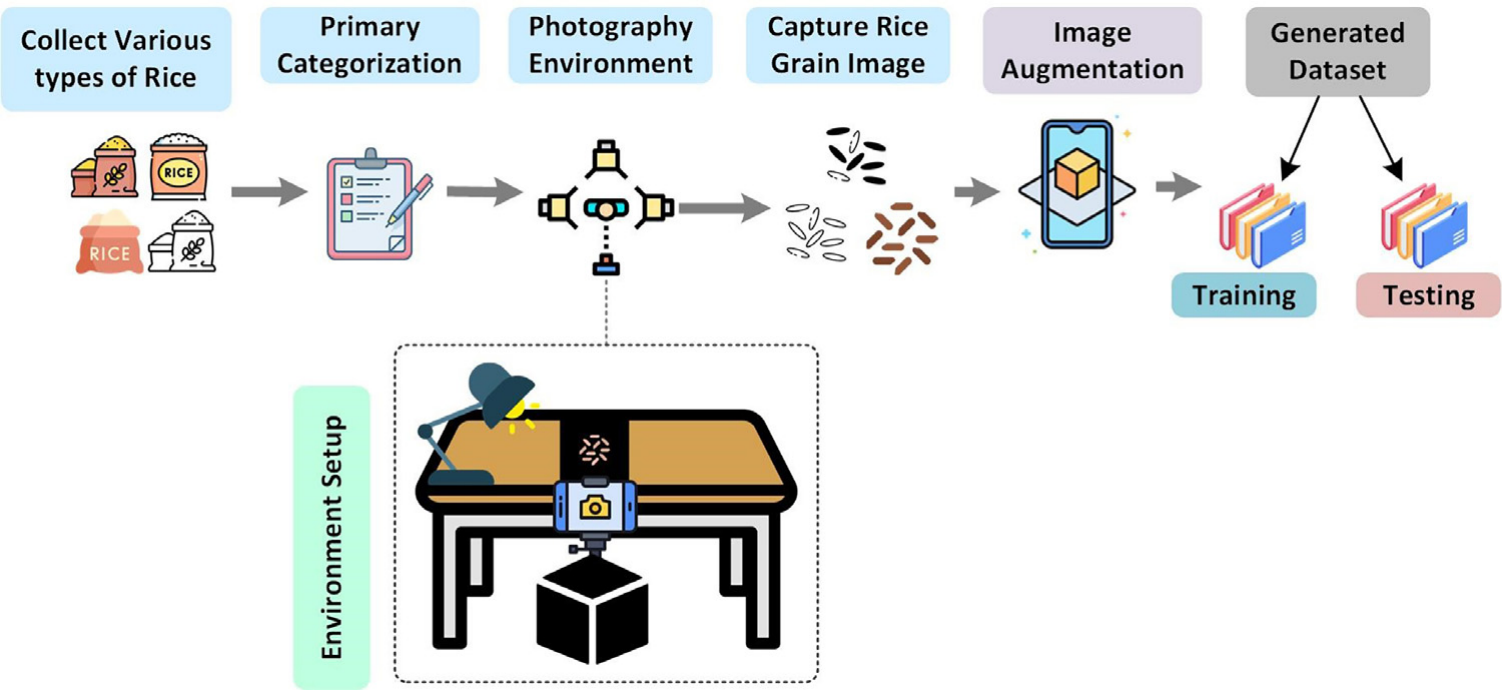
Workflow for the rice varieties image dataset generation.

The image acquisition process for each rice variety adhered to a methodically designed workflow, as illustrated in Fig. 2. This method involved a systematic selection of individual rice grains from each variety, ensuring an equitable distribution through a uniform selection process. This strategy played a pivotal role in preserving a diverse and representative dataset. To achieve this diversity, a randomized selection process was implemented, where each rice grain chosen for photography was randomly selected from within a cluster of rice. We gathered 20 primary types of rice from various marketplaces and rural areas, ensuring their authenticity by consulting a domain expert. Subsequently, we stored the rice in distinct containers under controlled temperatures to prevent deterioration. To precisely identify each rice variety, we needed to closely examine individual grains, observing their color, texture, and shape. This led us to position our photography setup approximately 4.2 inches above the rice platform, employing a 5x zoom on the camera to capture detailed images of the grains. To ensure clarity, we maintained a black background for each rice grain and took multiple photographs from various angles.

Following the capture of images for each rice variety, these images were then transferred from the smartphone's internal memory to an external hard drive. These images were then scrutinized by a domain expert and meticulously categorized into separate folders. After, these images were meticulously organized into folders, with each folder labeled with the corresponding rice variety. The image acquisition process for the subsequent rice type began once the images of the previously captured rice type were removed from the smartphone's memory. This systematic and iterative approach continued until images for all rice categories were successfully obtained. To address the substantial image volume demands of deep learning models for machine vision, data augmentation techniques were employed in our raw image data. Rotations of 45°, 90°, and 180°, along with horizontal and vertical flips with a probability of 1, were applied automatically, generating an additional set of 23,650 augmented images. This significantly increases the dataset size and diversity, enhancing model generalizability and performance. To ease the experimenting process for the researchers we have balanced the data and split it in an 80:20 train-test ratio. The ‘Train_n_Test.zip’ folder contains two sub-directories: ‘1_TEST’ which contains 1125 images per class and ‘2_VALID’ which contains 225 images per class.

### 4.1 Camera specification

All images were taken with an iPhone 11 camera. This smartphone has a dual-camera system featuring a 12MP ultra-wide lens and a 12MP Wide lens. The ultra-wide lens, with an ƒ/2.4 aperture and a broad 120° field of view, complements the wide lens with its ƒ/1.8 aperture. The camera setup includes 2x optical zoom out and digital zoom capabilities up to 5x. Table 3, shows the details camera setup.

**Table 3:** Camera Setup Details.

Wide Camera	26mm
Focal Length (*f*)	1.8
Brightness	Studio Light
Resolution	9MP
ISO	125, 135 mm, −2 *ev*, 1/49 *s,*
Zoom	5x
Camera Distance from Object	4.2 inchs (approx.)

## Limitations

The dataset has certain limitations. While it includes twenty distinct rice varieties, it may not encompass the full range of existing types, potentially limiting the model's adaptability to other varieties. Additionally, the dataset is specifically tailored to the Bangladeshi local marketplace, meaning that rice names might differ in other regions. It's important to note that this dataset primarily comprises images of processed or semi-processed rice; therefore, tasks related to raw rice classification may encounter challenges.

## Ethics Statement

This article does not involve any research involving human or animal subjects by any of the authors. The datasets utilized in this article are publicly accessible. When utilizing these datasets, it is essential to adhere to appropriate citation guidelines.

## CRediT authorship contribution statement

**Md. Masudul Islam:** Conceptualization, Investigation, Software, Validation, Formal analysis, Methodology, Resources, Data curation, Writing – original draft, Visualization. **Galib Muhammad Shahriar Himel:** Methodology, Formal analysis, Resources, Writing – original draft, Writing – review & editing. **Mohammad Shorif Uddin:** Supervision, Writing – review & editing. **Md. Golam Moazzam:** Supervision, Validation, Writing – review & editing.

## Data Availability

An Image Dataset of Rice Varieties (Original data) (Mendeley Data).
